# Identification of divergent *Leishmania* (*Viannia*) *braziliensis* ecotypes derived from a geographically restricted area through whole genome analysis

**DOI:** 10.1371/journal.pntd.0007382

**Published:** 2019-06-06

**Authors:** Bruna S. L. Figueiredo de Sá, Antonio M. Rezende, Osvaldo P. de Melo Neto, Maria Edileuza F. de Brito, Sinval P. Brandão Filho

**Affiliations:** 1 Department of Microbiology, Aggeu Magalhães Institute/FIOCRUZ, Recife, Pernambuco, Brazil; 2 Department of Immunology, Aggeu Magalhães Institute -FIOCRUZ, Recife, Pernambuco, Brazil; Universiteit Antwerpen, BELGIUM

## Abstract

*Leishmania braziliensis*, the main etiological agent of cutaneous leishmaniasis (CL) in Latin America, is characterized by major differences in basic biology in comparison with better-known *Leishmania* species. It is also associated with a high phenotypic and possibly genetic diversity that need to be more adequately defined. Here we used whole genome sequences to evaluate the genetic diversity of ten *L*. *braziliensis* isolates from a CL endemic area from Northeastern Brazil, previously classified by Multi Locus Enzyme Electrophoresis (MLEE) into ten distinct zymodemes. These sequences were first mapped using the *L*. *braziliensis* M2904 reference genome followed by identification of Single Nucleotide Polymorphisms (SNPs). A substantial level of diversity was observed when compared with the reference genome, with SNP counts ranging from ~95,000 to ~131,000 for the different isolates. When the genome data was used to infer relationship between isolates, those belonging to zymodemes Z72/Z75, recovered from forested environments, were found to cluster separately from the others, generally associated with more urban environments. Among the remaining isolates, those from zymodemes Z74/Z106 were also found to form a separate group. Phylogenetic analyses were also performed using Multi-Locus Sequence Analysis from genes coding for four metabolic enzymes used for MLEE as well as the gene sequence coding for the Hsp70 heat shock protein. All 10 isolates were firmly identified as *L*. *braziliensis*, including the zymodeme Z26 isolate previously classified as *Leishmania shawi*, with the clustering into three groups confirmed. Aneuploidy was also investigated but found in general restricted to chromosome 31, with a single isolate, from zymodeme Z27, characterized by extra copies for other chromosomes. Noteworthy, both Z72 and Z75 isolates are characterized by a much reduced heterozygosity. Our data is consistent with the existence of distinct evolutionary groups in the restricted area sampled and a substantial genetic diversity within *L*. *braziliensis*.

## Author summary

*Leishmania braziliensis* is the main etiological agent of cutaneous leishmaniasis (CL) in Latin America. In the Pernambuco state, a highly endemic area for CL in Northeastern Brazil, ten zymodemes have been previously described, identified through the detection of variations in the mobility of selected enzymes on gel electrophoresis (MLEE). Here we used next-generation genome sequencing to reevaluate the genetic diversity and relatedness between isolates representing these zymodemes. The genetic analysis clustered these isolates into three distinct phylogenetic groups, with those circulating in forested environments more related to each other (Z72 and Z75) and separated from the other eight isolates from more urbanized environments, but nevertheless forming two further groups. All ten isolates were definitely identified as *L*. *braziliensis*, including one from zymodeme Z26, previously identified as *L*. *shawi* through MLEE. Chromosome copy number variation was observed in all isolates studied, but restricted mainly to chromosome 31, with a single isolate (from zymodeme Z27) showing more than two copies of other chromosomes. A low heterozygosity observed only for both Z72/Z75 isolates also confirm differences associated with them. Our findings confirm that MLEE, despite still being considered the gold standard for characterization of *Leishmania* spp., does not reflect relevant information on intra-specific variations. It also highlights the substantial diversity observed between *L*. *braziliensis* isolates.

## Introduction

Cutaneous Leishmaniasis (CL) is an infectious parasitic disease characterized by a very dynamic eco-epidemiology, which is associated with several cycles of transmission and involves a variety of reservoirs and competent vectors that vary according to different geographical regions. *Leishmania* (*Viannia*) *braziliensis* is the major *Leishmania* species responsible for CL in Brazil, as well as in several Central and South American countries (from Belize to Argentina), all of which are likely not to correspond exactly to the same parasite species but subspecies complex [1–3]. Intra-specific diversity has been described in Brazil [4–5], even within a single federal state [6], and it is possible that the different transmission cycles collaborate for the generation and maintenance of this diversity [5]. The existing variability may favor survival in diverse ecological systems and may also determine the distribution of the observed clinical forms of the disease [7, 8].

The entire eco-epidemiological context for the *L*. *braziliensis* CL linked to the diversity of the parasite, a reflex of its genetic plasticity [9–10], needs to be elucidated, mainly with the aid of genomic tools. Some studies have shown aneuploidy as one of the main evolutionary strategies of these parasites for adaptation to environmental modifications and resistance to drugs [11–13]. Comparisons between genomes of *Leishmania* species confirmed that despite evolutionary divergences within the genus, there is a high degree of synteny. The subgenera *Leishmania* and *Viannia* are characterized by a large conservation of gene sequences with few species-specific genes or paralog groups. Nevertheless, it is known that *L*. *braziliensis* has some peculiarities in its genome that are absent from the Old World *Leishmania* species, such as transposable elements and genes encoding the RNA interference (RNAi) machinery [14–17]. The presence of a virus called *Leishmania* virus 1 (LRV1) inside the *Leishmania* parasite has been related to variations in disease manifestation. Indeed, in animal models, it has been demonstrated that the presence of double-stranded RNA that characterizes the virus stimulates an exacerbated immune response, with lesions characteristic of the mucosal form of the disease [18, 19]. *Leishmania* RNA viruses were identified and characterized for several years in *L*. (*Viannia*) *braziliensis* and *L*. *guyanensis* [20–22] as well as in a single *L*. *major* isolate [23].

*Leishmania* parasites constitute a complex biological model from an ecological, genetic and phylogenetic point of view [24]. In fact, despite considerable progress in the study of their cellular and molecular biology, as well as their evolutionary genetics, there are still many unknown parameters that need to be appreciated to better understand the biology of *Leishmania* species, such as the extent to which genetic exchange may impact on their overall diversity. *Leishmania* species are capable of a meiotic sex cycle within the insect vector, producing hybrids that have complete genomic fragments of both parents, but the mitochondrial maxicircle (kDNA) of only one parent [25–27]. Mating occurs only in the vector, and hybrids can be transmitted to the mammalian host, which confirms the strong contribution of this mode of genetic exchange to phenotypic diversity in natural populations.

In the Northeastern region of Brazil, several aspects related to the eco-epidemiology of CL caused by *L*. *braziliensis* in the Atlantic Rain Forest region have been investigated in the last twenty years. These studies were based on a large number of isolates recovered from human patients, from wild and synanthropic rodents (*Necromys lasiurus*, *Nectomys squamipes*, *Rattus rattus*) and from the sandfly vector *Lutzomyia whitmani* [28, 29], all from the State of Pernambuco. These isolates were originally classified with a panel of monoclonal antibodies and found to be compatible with *L*. *braziliensis*. They were also characterized using a phenotypic analysis, MLEE (Multi Locus Enzyme Electrophoresis), which is the gold standard for identification of *Leishmania* spp. MLEE confirmed the occurrence of 10 different zymodemes, with nine of those (72 isolates) corresponding to *L*. *braziliensis* and one zymodeme corresponding to *Leishmania shawi* (five isolates), which was an unprecedented finding in this region [29]. A large phenotypic heterogeneity was thus suggested for these isolates, contrasting with the reduced geographic area from which they were recovered [28–33].

In view of the recent advances in genome sequencing technology, and considering that proper phylogenetic analyses and relationships can be best inferred from comparisons of whole genome sequences, the present study aimed to evaluate the genetic variability of selected isolates of *L*. *braziliensis* from the State of Pernambuco using for the first time a whole genome sequencing approach. One representative isolate from each of the ten zymodemes identified was thus selected for next generation sequencing. Based on the comparison of the ten genomes and including selected sequences from publicly available databases, several analyses were carried out. Phylogenetic analyses were first performed based on comparisons of single nucleotide polymorphisms (SNPs) and sequences of selected genetic markers, and these were followed by heterozygosity and ploidy determination. This study allowed a high resolution investigation on the variability of these isolates, adding information regarding their relationship to one another as well as to other *L*. *braziliensis* isolates and *Leishmania* species. The genetic analysis grouped these isolates into three distinct phylogenetic groups, with isolates circulating in forested environments more related to each other and separated from the remaining eight isolates from more urbanized environments, but nevertheless forming two further groups. Distinct evolutionary groups were then found in the restricted area sampled, highlighting the greater genetic diversity within *L*. *braziliensis* when compared with previously studied *Leishmania* species.

## Methods

### Samples and cell culture

Each *L*. *braziliensis* isolate selected for this study was previously classified in a zymodeme by the Laboratory of Leishmaniasis Research—IOC, FIOCRUZ-RJ [29], followed by storage in liquid nitrogen at the Institute Aggeu Magalhães/FIOCRUZ. When live cells were needed, they were recovered from the frozen stock. Samples were thawed and maintained in Schneider’s pH 7.2 medium, supplemented with 10% Fetal Bovine Serum (SFB), at 25 °C ± 1°C in an incubator and subsequently expanded when needed.

### Genome sequencing

DNA extraction for the *L*. *braziliensis* isolates was carried out using the QIAamp DNA Mini Kit (Quaigen). DNA quantification was performed using Nano Drop 2000C (Thermo Fischer) and Qubit (Life Technologies). For preparation of the paired-end libraries, the NexteraXT DNA kit (Illumina) was used as recommended by the manufacturer with 1 ng of genomic DNA for each sample. Paired-end libraries were sequenced using: MiSeq Reagent Kit v2 (500-cycles); MiSeq Reagent Kit V3 (300 cycles); and Miseq Reagent Kit V3 (600 cycles). After sequencing, the quality of the data generated in the MiSeq System Illumina platform was verified through the FastQC (v.0.11.5) quality control tool (https://www.bioinformatics.babraham.ac.uk/projects/fastqc/) [34]. The total number of generated reads from each sample and parameters such as read size and quality per sequenced base were evaluated (see S1A and S1B Fig) and also GC content. Bases with low quality (Q<30) were removed using the Trimmomatic program (version V.30) [35] applying the following parameters: -phred33, ILLUMINACLIP:AdapterFile.fasta:2:30:10, LEADING:20,TRAILING:20, SLIDINGWINDOW:5:20 and MINLEN:150. The sequences of the ten new *L*. *braziliensis* genomes were deposited in the Sequence Read Archive-SRA on Submission (SUB4143783) and in the NCBI BioProject (PRJNA475480).

### Mapping, SNP annotation and processing

The reads from the genomes sequenced here were mapped against the reference *L*. *braziliensis* genome (MHOM/BR/75/M2904) using the BWA-MEM algorithm from the BWA package, version 0.7.10 [36]. Thus, one BAM file was generated for each of the 10 sequenced genome samples. In order to evaluate the performance of the mapping step, the algorithm CollectAlignmentSummaryMetrics from Picard package was executed (S1 Table summarizes the resulting alignment metrics). Next, using the HaplotypeCaller algorithm from the GATK package, version 4.0.8.1 [37], the BAM files plus the FASTA file of the reference genome were compared to call SNPs. To perform a cohort analysis workflow, the parameter *ERC* was set to *GVCF* (Genomic VCF), producing reference confidence values condensed in non-variant blocks. Single VCF files were thus generated for each genome sample which were then integrated using the GenomicsDBImport tool. After integration, one VCF file was produced for each *L*. *braziliensis* chromosome although each file had the integrated information of the 10 samples. The extraction of genetic diversity for each chromosome was then performed using the GenotypeGVCFs tool. All genetic variations found from the chromosome VCFs were filtered by quality parameters (QD <2.0, FS>60, MQ<40, MQRankSum < -12.5, ReadPosRankSum < -8.0) with VariantFiltration and applying the SelectVariants tool to select only SNPs as genetic variations, the last four tools also being part of the GATK package. The annotation of the variations found was done using the snpEFF software, version 4.3, which classifies SNP effects according to an impact category (low- synonymous changes; moderate- codon change/deletion/insertion; high- frame shifts, addition/deletion of stop codons), localization in the genome and functional class [38]. The MultiQC tool (v1.0.dev0) [39] was used to summarize the outputs generated by SnpEff.

In order to perform a broad comparative analysis based on the VCF files, the VCFTools package (V.4.0) was used, with the—*diff* and—*diff-site* parameters, to calculate the number of shared SNPs for each pair of the 10 genome samples. A quadratic distance matrix was then generated and used as input for the *neighbor* algorithm of the PHYLIP package, version 3.696 [40], to create the dendrogram with the 10 samples. For the Principal Component Analysis [41], the VCFTools package was also applied with the parameter—*012*, allowing the program to generate a genotype matrix. The loci with missing data were then excluded and the matrix loaded to the R environment. PCA calculations and visualization were performed using the *prcomp* and *fviz_pca_ind* functions, respectively. In addition, the heterozygosity for each chromosome from each sample was calculated using the VCFs and the function *heterozygosity* from the SeqVarTools, within the R environment. The heterozygosity values were then logarithmically transformed and plotted.

### Phylogenetic and diversity analyses

The four metabolic enzymes used here for the phylogenetic analysis were chosen due to the fact that they are important markers for characterization of species from the subgenus *Viannia*: isocitrate dehydrogenase (ICD, EC 1.1.1.42- LbrM.33.2820); mannose phosphate isomerase (MPI, EC 5.3.1.8- LbrM.32.1750); 6-phospho gluconate dehydrogenase (6PGDH, EC 1.1.1.44- LbrM.34.3250); and glucose-6-phosphate dehydrogenase (G6PDH, EC 1.1.1.49- LbrM.20.0160). Sequences encoding all four enzymes were extracted from the 10 genomes sequenced here and from 64 *L*. *braziliensis* genomes (50 Peruvian and 14 Bolivian), part of a pre-publication release (NCBI BioProject: PRJEB4442) that could be used for the purposes of this study. The fastq-dump tool (https://ncbi.github.io/sra-tools/fastq-dump.html) was used to download all the sequence data (SAM format) from SRA. In addition, sequences from 93 isolates from different species of the subgenus *Viannia* (*L*. *braziliensis*, *L*. *guyanensis*, *L*. *laisoni*, *L*. *lindenbergi*, *L*. *shawi*, *L*. *naiffi* and *L*. *guyanensis*) [42] were also retrieved. For the Hsp70 (LbrM.28.2990) analysis, sequences from our 10 genomes and from the samples loaded from the SRA database were compared with 12 sequences of strains of the subgenus *Viannia* (*L*. *braziliensis*, *L*. *laisoni*, *L*. *panamensis*, *L*. *guyanensis* and *L*. *naiffi*), deposited in GenBank by Fraga et al. [43]. Thus, the gene sequences were extracted using first the tool SelectVariants, from the GATK package, with the parameter excludeNonVariants. These VCF files were then used as input for the tool FastaAlternateReferenceMaker, also from GATK, in order to produce the FASTA files for each gene sequence containing the variant sites.

For the MLSA analysis, the sequences from the four housekeeping genes from each sample were first concatenated in the following order: MPI, ICD, 6PGDH and G6PD. The concatenated sequences were then aligned using the MAFFT software [44] with the global multiple alignment mode. The sequences were cut with the Trimal tool so that we obtained the same size for all samples as 2902 pb. Then, to choose the evolutionary model that best explains the alignment profile, eleven substitution models were tested, including models with equal / unequal gene frequencies, models with / without a proportion of invariant sites and models with / without variation between sites. A total of 88 models were tested using jModelTeste 2.1.3 [45, 46]. The selected model was TPM3uf + I + G (p-inv = 0.8160, Gamma shape = 1.2820), for the analysis with the four genes mentioned above, and HKY+I (p-inv = 0.7744, Gamma shape = 0.0604) for Hsp70. Alignments were used as input to PhyML algorithm, version 3.1, to estimate the maximum likelihood phylogeny [40]. The resulting tree was viewed and edited using iTOL (interactive Tree Of Life) (https://itol.embl.de/itol.cgi) [47].

For the *in silico* analysis on the diversity of the MLEE protein panel, the SNP annotation data was analyzed focusing specifically on the coding regions for the 14 metabolic enzymes genes used for MLEE typing. Here we characterized the impacts (Low, Moderate and High) of the SNPs for the 14 genes present in the ten *L*. *braziliensis* genomes, using the data generated by the snpEFF software, version 3.6.

### Ploidy number definition

For the calculation of chromosome copy number, we followed the methodology of Zhang et al. [48]. Prior to that, we used the bamCoverage tool from the deepTools package (https://deeptools.readthedocs.io/en/develop/content/tools/bamCoverage.html), to estimate sequencing depth from each chromosome, and normalized using the RPKM method. The median depth of each chromosome (d_chr_) was then determined followed by calculation of the total median depth for all chromosomes (d_T_ = median[d_chr1_…d_chr35_]). These values were then divided by two in order to represent the median depth for a haploid allele of a chromosome. The chromosome copy number was given by S_chr_ = d_chr_/(d_T_/2). These steps were implemented in a R script to perform the analysis automatically. We assumed that in general all chromosomes were diploid.

## Results

### Isolate identification and overview of the genomic data

**Fig 1:**
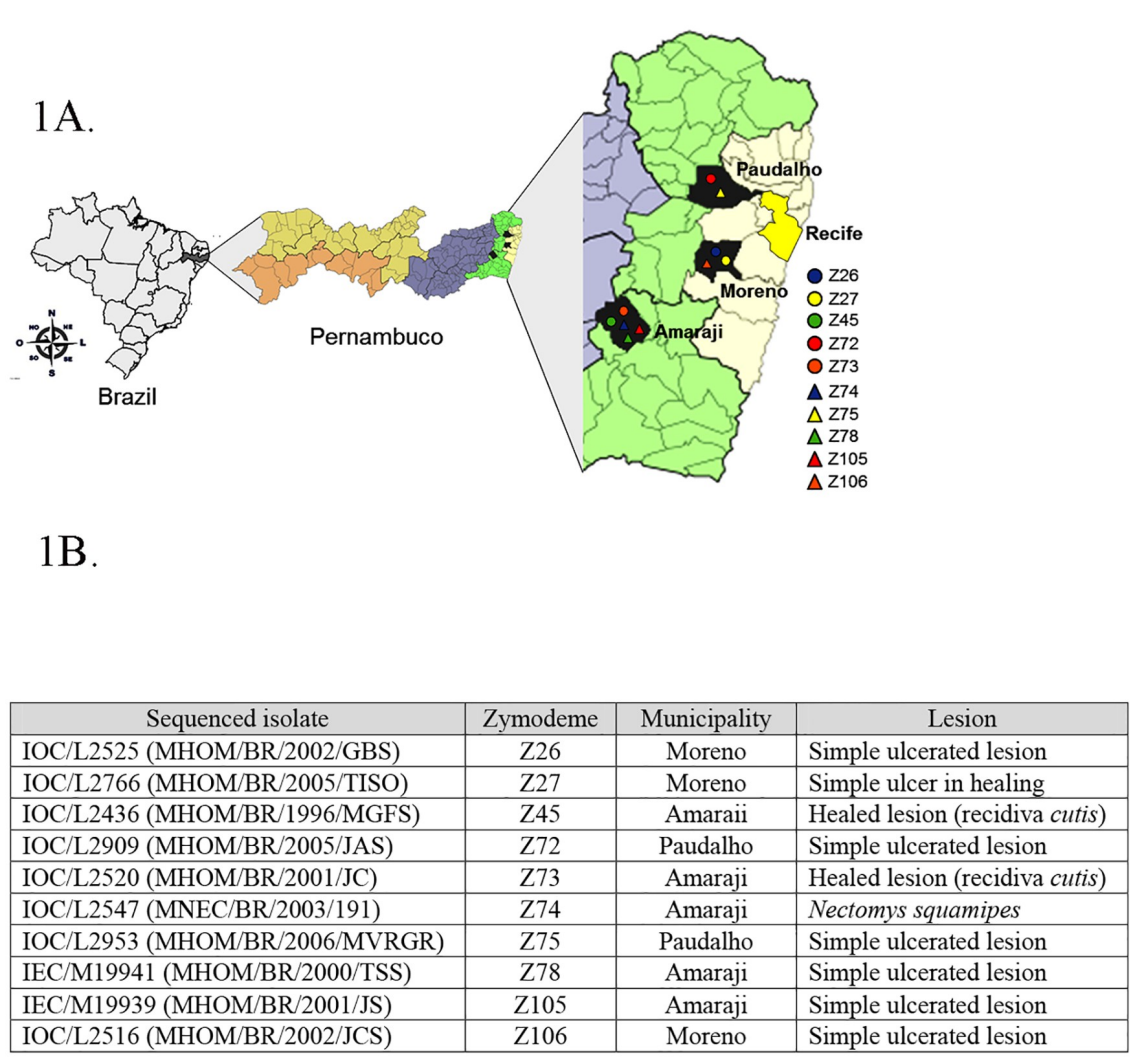
Overview of the isolates/zymodemes selected for this study. **A** Summarized description of the isolates investigated here, including the zymodemes they belong too. All isolates had their zymodemes (Z26, Z45, Z72, Z73, Z74, Z75, Z78, Z105 and Z106 in the figure) characterized by Multi Locus Enzyme Electrophoresis (MLEE). **B** Mapping the localizations from where the isolates studied here were found, representative of the ten zymodemes of *Leishmania* (*V*.) *braziliensis* from the state of Pernambuco, northeastern Brazil. The isolates are from three municipalities (Amaraji, Moreno and Paudalho) endemic for Cutaneous Leishmaniasis.

Based on the zymodeme profile of the previously investigated *L*. *braziliensis* isolates from Pernambuco, a large phenotypic diversity was recovered from a restricted geographic area within this state. It has been suggested that this genetic diversity may be associated with the complexity of *L*. *braziliensis* transmission, probably reflecting the parasite adaptation to different vector species [5,29,31]. The previous MLEE analysis, however, could not clearly define the true phylogenetic relationships between the various isolates, and major questions regarding their ecoepidemiology could not be properly answered without a better definition of these relationships. To solve this, representative isolates from the nine *L*. *braziliensis* zymodemes (IOC/Z27, IOC/Z45, IOC/Z72, IOC/Z73, IOC/Z74, IOC/Z75, IOC/Z78, IOC/Z105 and IOC/Z106) and a single isolate classified as a *L*. *shawi* zymodeme (IOC/Z26) were selected for this study for next generation sequencing and whole genome analyses. A single isolate for each zymodeme was chosen, with relevant features from each described in Fig 1A. These isolates were derived from three municipalities surrounding or within the Metropolitan Region of Recife, capital of the State of Pernambuco (see Fig 1B). For two of these municipalities, Amaraji and Moreno, the isolates were recovered from individuals infected within the peridomestic environment or from a rodent captured in similar surroundings. In contrast the infections from the third municipality, Paudalho, occurred within a heavily forested remnant of Atlantic Rain Forest.

Following the initial analyses and filtering steps of the raw sequencing data, mapping the reads of the 10 genomes was performed against the reference *L*. *braziliensis* genome (from the M2904 strain). After the quality control steps, the average genome sequencing depth ranged from six to nearly 30 fold, with the average length of the reads ranging from 188 to 211 bp, and the total number of paired reads per isolate ranging from 1,028,976 for the representative from zymodeme Z73, to 5,116,624 reads, for the Z106 isolate. These reads were then aligned against the reference genome, with the total number of aligned reads for the 10 genomes ranging from 1,000,624 (Z73) to 4,554,467 (Z106) and the percentage of unaligned reads falling between 1.5 (Z78) and 10.9% (Z106). The S1 Table summarizes the mapping statistics for all ten genomes described here.

### SNP counts and evaluation of genetic diversity

**Fig 2:**
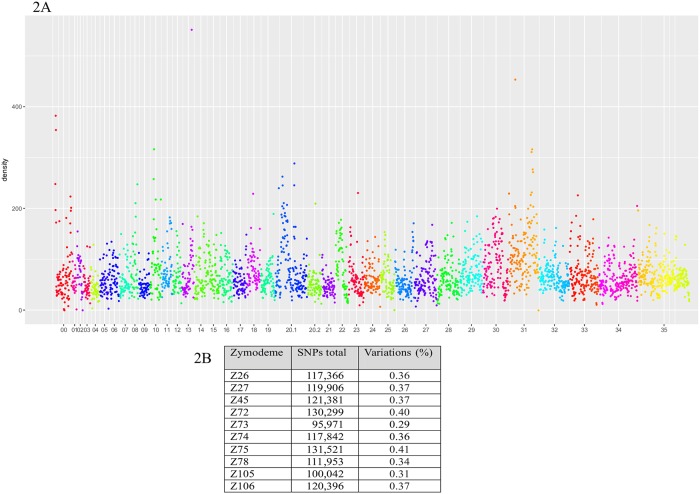
Overview of the SNP identification results. **A** Density of SNPs per chromosome for every 10 kb of the ten genomes of *L*. *braziliensis* sequenced here. **B** Total number of SNPs and percentage of variation observed between each of the new *L*. *braziliensis* genomes here and the reference genome from *L*. *braziliensis* M2904.

After mapping the reads against the reference genome, we performed Single Nucleotide Polymorphism (SNP) identification and annotation for all ten genomes sequenced here, again in comparison with the reference *L*. *braziliensis* M2904 genome. First, considering that differences in genome sequencing depth, annotation and SNP calling methods may lead to changes in the overall SNP numbers, we analyzed the density of SNPs along these genomes using a 10 kb window. As shown in Fig 2A, it is possible to observe that the SNPs are in general distributed homogeneously throughout the genomes. In addition, we plotted the sequencing depth for all chromosomes of *L*. *braziliensis* in 10 Kb windows (S2 Fig), confirming that the depth of sequencing was also homogeneous, except for chromosomes 31 and 35 (for chromosome 31 this is probably due to the greater number of copies seen for this chromosome). Most of the SNPs identified here were found to occur upstream or downstream of coding regions. The sum of the effects of low and moderate impact was roughly 10%, for all sequenced genomes. Regarding the functional class of the effects, half of them were classified as silent and the other half as missense mutations.

Fig 2B summarizes the overall diversity observed in SNPs between the sequenced isolates and the reference genome. This diversity is considerably large, with the number of SNPs ranging from ~96,000, for the least divergent isolate, representative of the Z73 zymodeme, to ~132,000 SNPs, for the most divergent Z75 isolate. For comparison, the number of SNP variants between two *L*. *peruviana* isolates sequenced recently and the *L*. *braziliensis* M2904 genome range from ~137,000 to ~144,000 [49], and much less variation has been seen with species belonging to the subgenus *Leishmania*, with SNP counts between distinct *L*. *donovani* and *L*. *infantum* isolates and the reference genomes falling below 4,000 [50,51]. Indeed, even between *L*. *amazonensis* and *L*. *infantum* the number of SNP variants identified seem to be much lower than those observed here for the different *L*. *braziliensis* isolates, with counts ranging between ~18,000 and ~24,000 SNPs [52]. These results indicate a much greater variability within *L*. *braziliensis* and which is even more relevant considering the variability observed between isolates from very near or adjacent geographical areas.

### Grouping and phylogenetic inferences based on the SNP analysis

Next, to determine which polymorphisms individual isolates share, pair-wise comparisons were performed using the 10 genomes sequenced here. The number of SNPs which two isolates share and the percentage of shared SNPs from these genomes were calculated and compared. For the Z72 and Z75 isolates, the most divergents when compared with the reference genome, with ~130,000 and ~132,000 SNP variants found, respectively, these isolates had the highest percentage of shared SNPs for any two isolates (~128,000 SNPs or 95.8%). Indeed this number of shared SNPs is similar to the ~128,000 SNP variants reportedly shared by the *L*. *peruviana* isolates, again in comparison with the reference *L*. *braziliensis* [49]. In contrast, both Z72/Z75 share much less SNP variants with any of the remaining isolates sequenced here, ranging between ~40,000 and ~48,000 and with the percentage of shared SNPs falling between 21 to 23%. Two other isolates, from zymodemes Z74 and Z106, also show very similar SNP profiles with ~114,000 shared variants in comparison with the reference genome, corresponding to 92.8% of shared SNPs. When compared with the remaining isolates (representatives of zymodemes Z26, Z27, Z45, Z73, Z78, Z105) the Z74/Z106 pair is also divergent, with the percentage of shared SNPs ranging from 57 to 65% and the number of shared SNP variants ranging from ~78,000 to ~95,000. Indeed the last six isolates are more closely related, and they seem to group together, although being overall more diverse, with the percentage of shared SNPs between any two isolates ranging from 72 to 93% (number of variants in common ranging from ~82,000 to ~116,000). Overall this data is compatible with three distinct evolutionary groups of *L*. *braziliensis* found among the isolates selected for this study.

**Fig 3:**
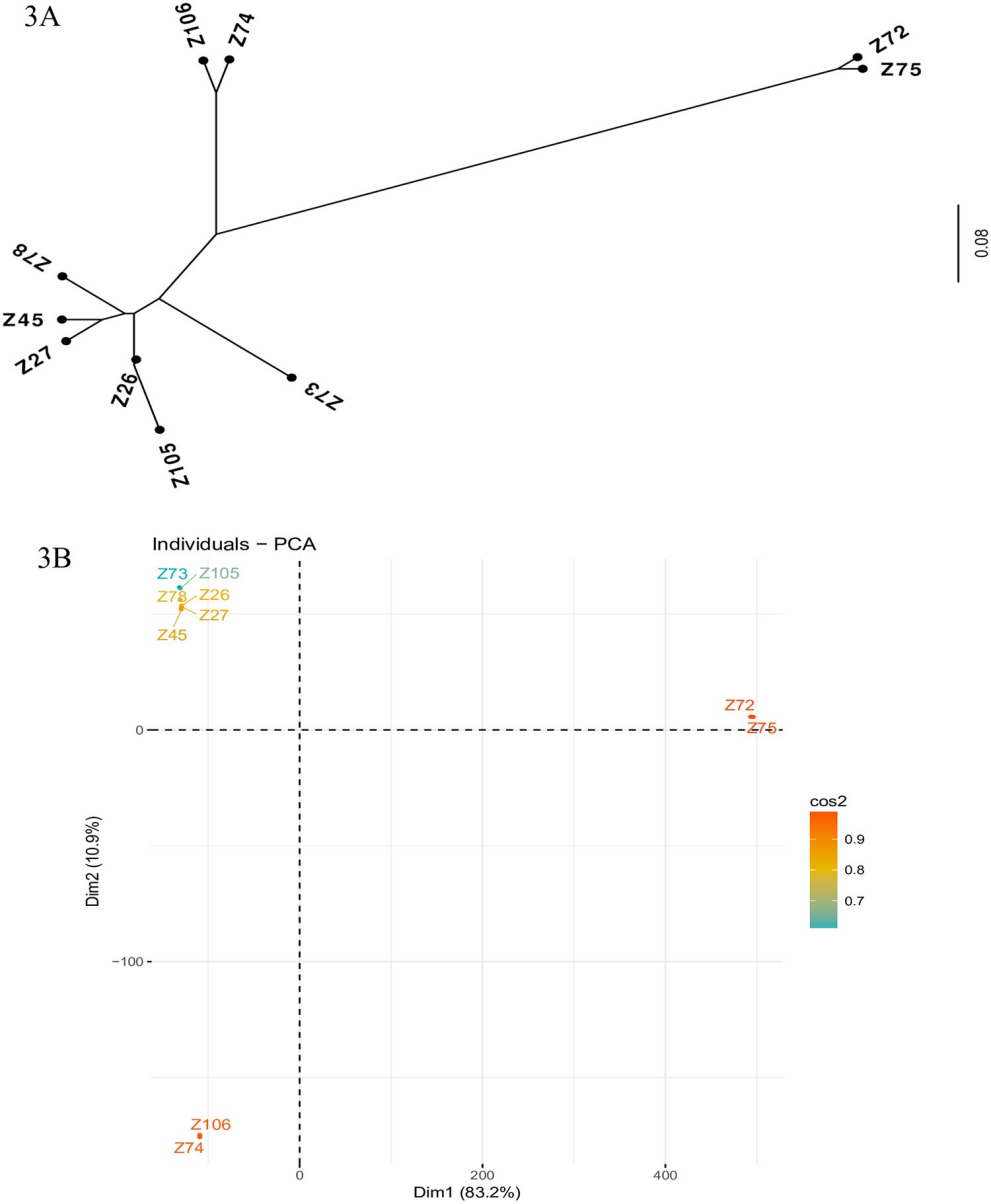
Assessment of the phylogenetic relationship between the *L*. *braziliensis* isolates evaluated here. A Phylogenetic tree built based on a distance matrix derived from SNPs shared among the ten isolates of *L*. *braziliensis* whose genomes were sequenced in this study (from zymodemes Z26, Z45, Z72, Z73, Z74, Z75, Z78, Z105 and Z106). **B** Principal Component Analysis (PCA) comparing the data from these ten genomes.

To better visualize the relationship between the different isolates sequenced here based on shared SNPs, a phylogenetic tree was built using a shared SNP matrix. As shown in Fig 3A, the Z72 and Z75 isolates group together and are well separated from the remaining isolates, although the Z74 and Z106 isolates also form a distinct subgroup. This same profile was found with a Principal Component Analysis (PCA) comparing the same isolates (Fig 3B), which show a distinct clustering of the Z72/Z75 pair and also group the Z74 and Z106 isolates separately. Considering these distinct groups, we decided to check if they could be correlated with the geographical areas from where they were isolated (see Fig 1A). Indeed the Z72 and Z75 isolates studied here are the only ones isolated from patients living in or infected within a forested area from the municipality of Paudalho. All other isolates were retrieved from the remaining two municipalities, and they are more associated with semi-urban and/or peri-domestic areas. It is noteworthy the fact that the Z74 isolate was the only one among the samples sequenced here which was retrieved from a rodent reservoir, while all others were isolated from infected human patients. Since all other isolates were derived from patients with single lesions, no correlation could be established between disease virulence and the genetic profile of the isolates.

### Phylogeny based on Multi-Locus Sequence Analysis and HSP70 sequence

**Fig 4:**
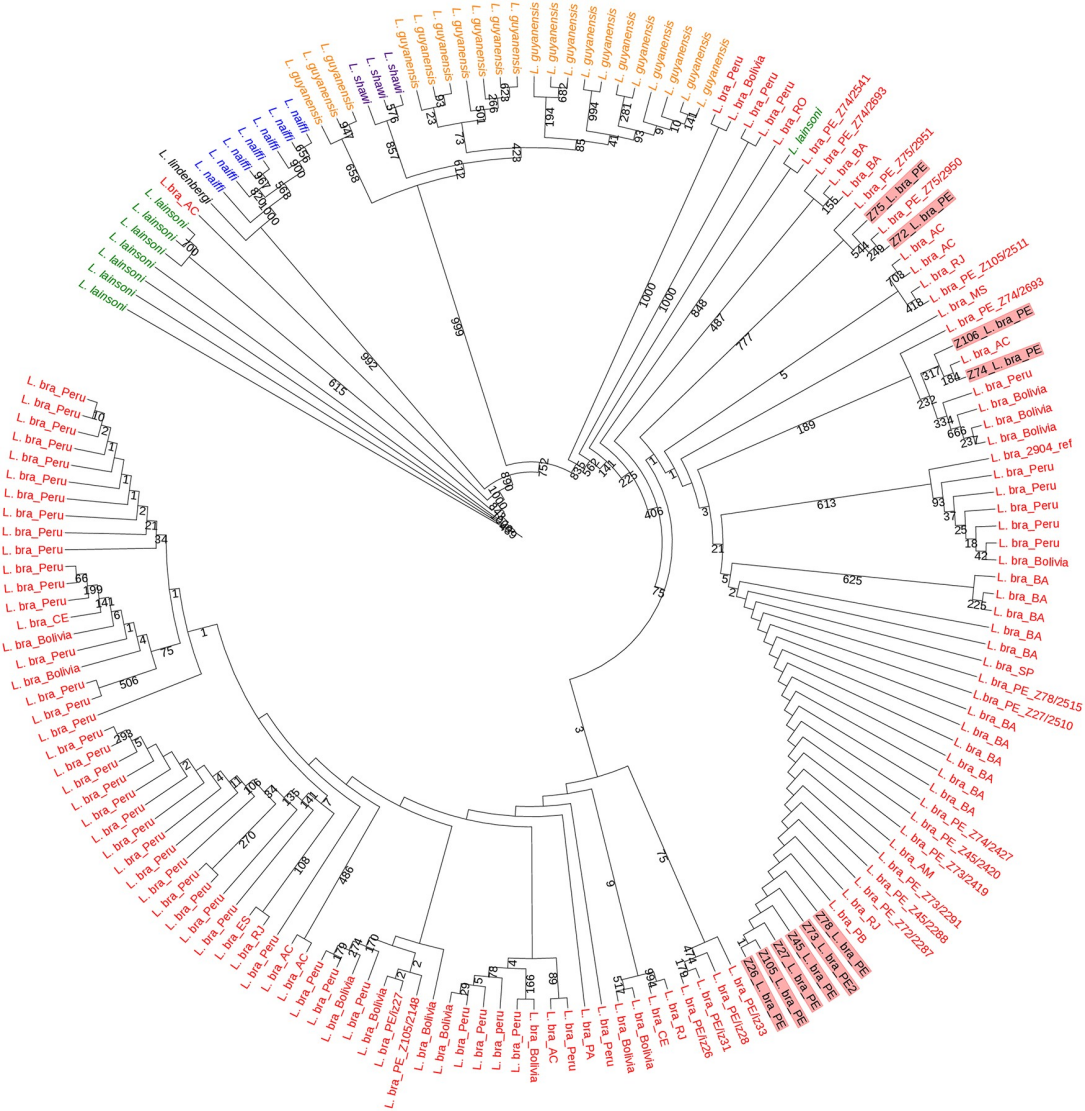
Phylogenetic tree based on the alignment of the sequences encoding four isoenzymes (ICD, MPI, 6PGDH and G6PDH) used to evaluate the genetic relationship between selected *L*. *braziliensis* isolates and those sequenced here. This analysis includes sequences from a total of 167 strains or lineages derived from 64 *L*. *braziliensis* genomes (Peruvian and Bolivian strains deposited in Sanger Institute—SRA), 93 samples from related species of the subgenus *Viannia* [42] and the 10 genomes of *L*. *braziliensis* sequenced here. The *L*. *braziliensis* M2904 strain was included as a control. The tree construction estimated the phylogeny by Maximum Likelihood, with a bootstrap of 1000 replicates. Brazilian states from where the *L*. *braziliensis* samples were isolated: AC—Acre; AM—Amazonas; BA—Bahia; CE—Ceará; ES—Espírito Santo; MS—Mato Grosso; PA—Pará; PB—Paraíba; PE—Pernambuco; RJ—Rio de Janeiro; RO—Rondônia; SP—São Paulo.

**Fig 5:**
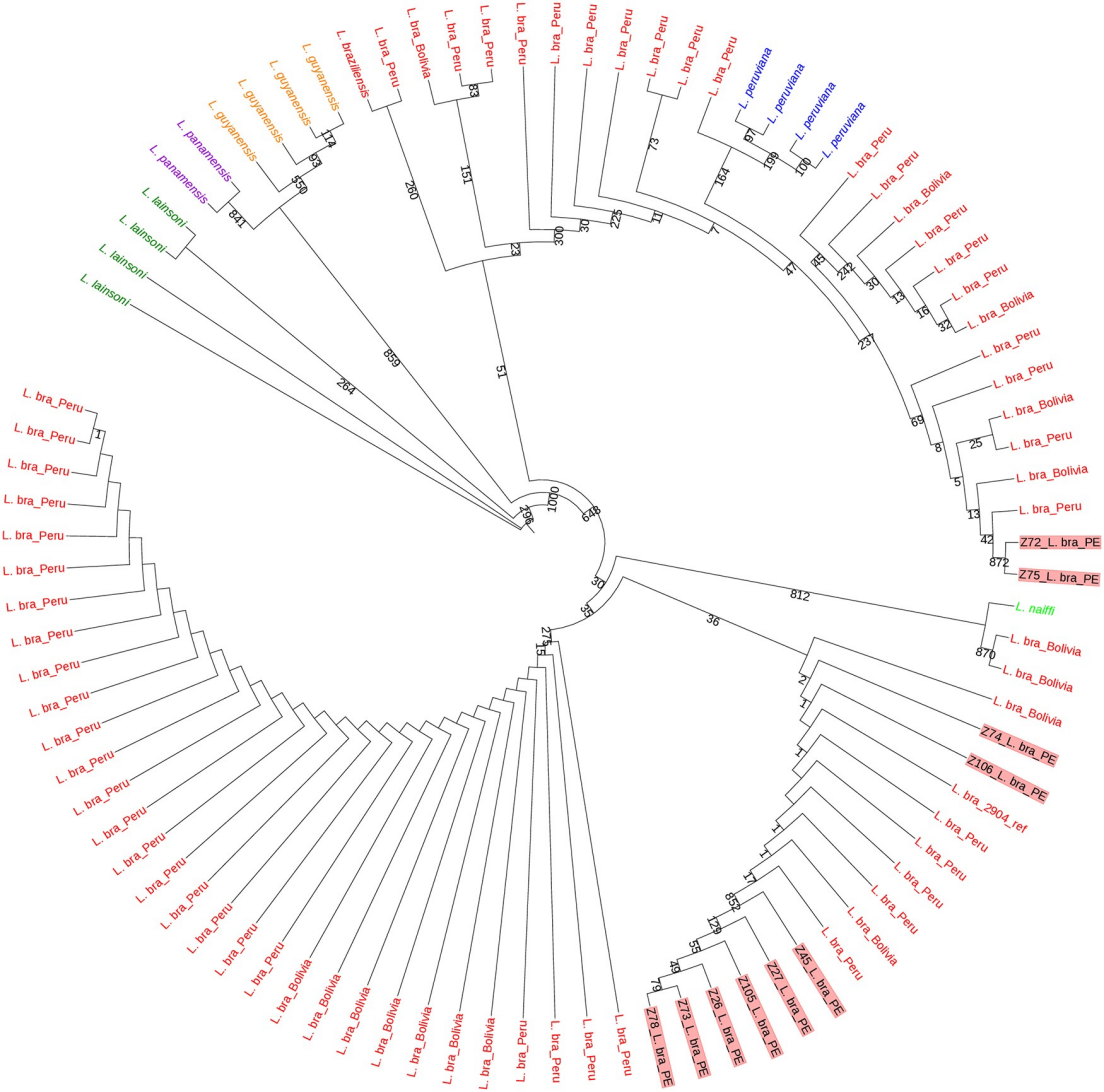
Phylogenetic tree generated from the alignment of the coding region of Hsp70. Sequences from a total of 91 strains were analyzed, derived from the 64 *L*. *braziliensis* genomes used in the previous figure plus 12 strains from related species of the subgenus *Viannia* [43] and the 10 genomes of *L*. *braziliensis* sequenced here, as well as the M2904 strain used as control. The tree construction estimated the phylogeny by Maximum Likelihood, with a bootstrap of 1000 replicates.

In order to infer the phylogenetic relationship between the isolates studied here and other *L*. *braziliensis* strains isolated elsewhere, we first retrieved, from their genomic sequencing data, the sequences encoding four metabolic enzymes used for the MLEE analysis (ICD—Isocitrate Dehydrogenase, MPI—Mannose Phosphate Isomerase, 6PGDH—6-Phospho-Gluconate Dehydrogenase and G6PDH—Glucose-6-Phosphate Dehydrogenase). Previously, partial sequences of the genes encoding these four enzymes were used to evaluate phylogenetic relationships between a large dataset of *L*. *braziliensis* isolates and related species belonging to the subgenus *Viannia* from different regions of Latin America. The four genes, representing different loci, were seen to have different degrees of diversity between groups of species and were found to be suitable to be used in combination for intra- and inter-specific inferences [42]. Sequences encoding all four enzymes were extracted from the 10 genomes sequenced here and from 64 *L*. *braziliensis* genomes (50 Peruvian and 14 Bolivian) that are part of a pre-publication release (NCBI BioProject: PRJEB4442). Equivalent fragments encoding the four enzymes were then used to perform a Multi-Locus Sequence Analysis (MLSA) comparing the ten newly sequenced genomes with the previously published dataset sequences (*L*. *braziliensis* from the Sanger Institute SRA—https://www.ncbi.nlm.nih.gov/sra) and with sequences from species of the subgenus *Viannia* (*L*. *braziliensis*, *L*. *guyanensis*, *L*. *laisoni*, *L*. *lindenbergi*, *L*. *shawi* and *L*. *naiffi*) deposited in GenBank (www.ncbi.nlm.nih.gov/Genbank) by Boité et al. [42]. The phylogenetic tree built based on this analysis is shown in Fig 4. Four distinct monophyletic groups are clearly identified, separating most of the *L*. *lainsoni* and *L*. *braziliensis* species in distinct clades and grouping with strong bootstraps *L*. *lindenbergi* with *L*. *naiffi* and *L*. *guyanensis* with *L*. *shawi*. All ten isolates sequenced here were found within a *L*. *braziliensis* monophyletic group, including the zymodeme 26 isolate, which displayed a phenotype compatible with *L*. *shawi* through MLEE [29, 31]. The isolates from zymodemes Z72 and Z75 are clearly clustered together with high confidence, while the Z74 and Z106 pair and the remaining six isolates (Z26, Z27, Z45, Z73, Z78 and Z105) are also part of separate groups, although their grouping is not supported by strong bootstrap values. To independently confirm these results we carried out a second analysis comparing the sequences of the cytoplasmic Hsp70 heat shock protein from our isolates with those of a large pool of *Leishmania* species from the subgenus *Viannia* (Fig 5). For this analysis, the same sequences from the SRA plus 12 sequences of strains from the subgenus *Viannia* (*L*. *braziliensis*, *L*. *laisoni*, *L*. *panamensis*, *L*. *guyanensis*, *L*. *peruviana* and *L*. *naiffi*) deposited in Genbank by Fraga et al, 2010 [43] were used. The results confirm that all ten isolates cluster with *L*. *braziliensis* and *L*. *peruviana* isolates and group separately from other *Viannia* species such as *L*. *guyanensis*, *L*. *lainsoni* and *L*. *panamensis*. They also indicate a well supported clade for the Z72 and Z75 isolates, with strong bootstraps, with Z74 and Z106 placed in an uncertain position but also separated from the clade consisting of the other six isolates studied here, grouped with high confidence. Noteworthy is the placement of the single *L*. *naifi* sequence, grouped with two *L*. *braziliensis* samples and separated from all other sequences evaluated in the figure. Overall the results from both phylogenies confirm the clustering of the samples sequenced here in three distinct *L*. *braziliensis* groups.

### Evaluating the *in silico* MLEE profiles

**Fig 6:**
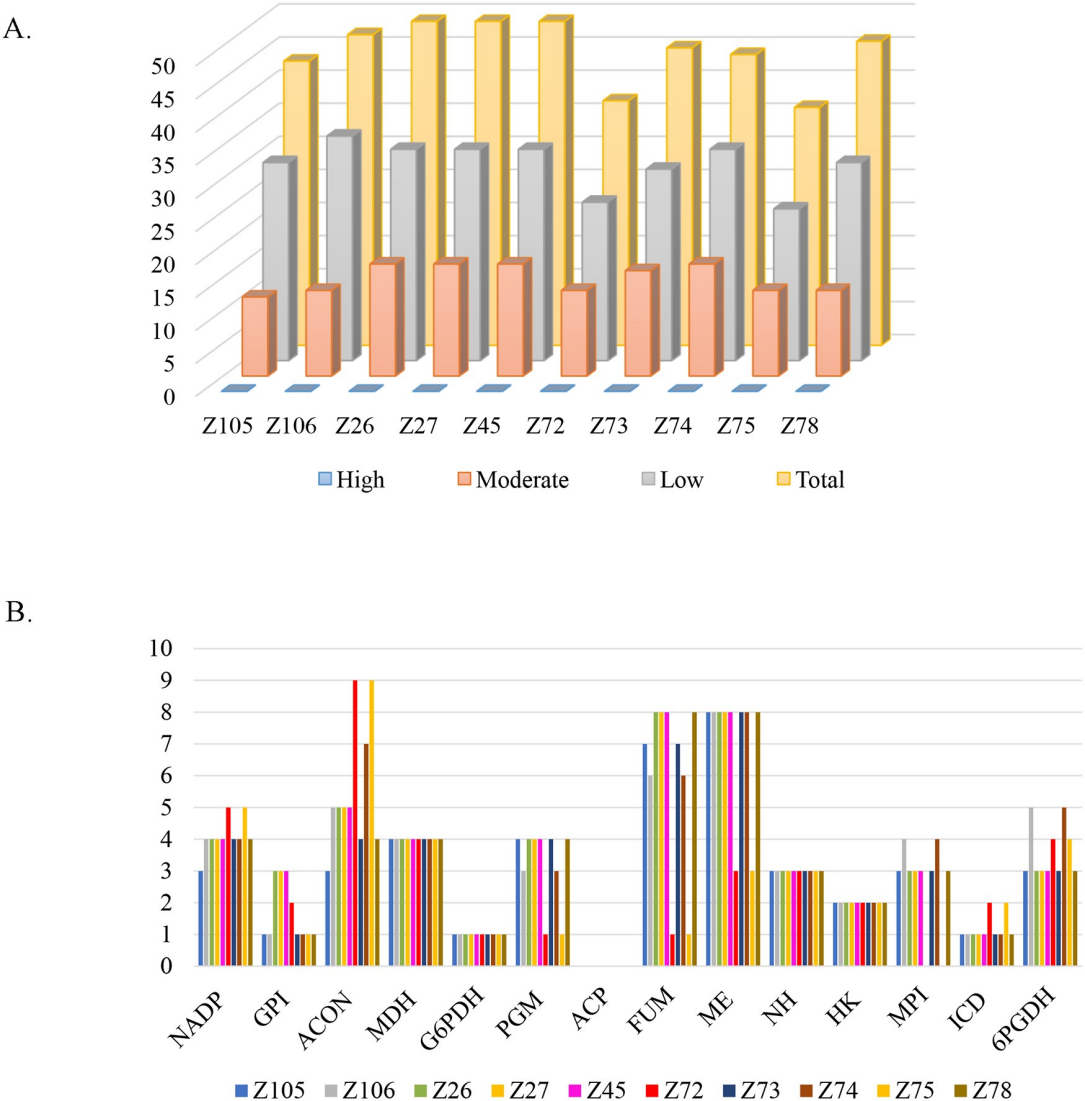
Assessment of the impact of the SNPs on the coding sequences of selected metabolic enzymes. **A**. Total SNPs with impact (low, moderate and high) in the coding region of the 14 metabolic enzymes (isoenzymes) analyzed for the ten targeted *L*. *braziliensis* genomes. **B**. Impact of SNP (low, moderate and high) on the coding sequence of individual enzymes (NADP, GPI, ACON, MDH, G6PDH, PGM, ACP, FUM, ME, NH, HK, MPI, ICD and 6PGDH).

The ability to characterize *Leishmania* species and identify their variants has major implications in assisting in the definition of a more effective treatment against the diseases they cause. MLEE is still considered the gold standard for this identification although many approaches have been developed to better contribute to the characterization of *Leishmania* species, such as MLSA- Multilocus Sequence Analysis [42, 53, 54]. Here, major differences were observed between the original MLEE classification in zymodemes for the ten isolates selected for this study [29] and the phylogenetic results derived from our genome analysis. For instance, the Z26 isolate, which by MLEE was classified as a separate species, was shown here to be very closely related to several other isolates and firmly clustered with *L*. *braziliensis*. In addition, no indication of the three separate *L*. *braziliensis* groups identified here could be derived from the MLEE analysis. In order to verify the reasons for these discrepancies, we decided to investigate the genetic basis for the different MLEE profiles observed for the ten isolates selected for this study. First, the full-length coding sequences were retrieved from 14 enzymes used for the MLEE panel (Table 1), and that could be identified for all the 10 genomes sequenced here, as well as the reference genome. A survey was then made of all SNPs causing amino acid changes in these enzymes, with these changes classified according to the impact they might have on the enzyme (low, moderate and high). A total of 298 low and 152 moderate impact SNPs were registered, with no SNP with high impact found for any of the 10 isolates. The isolates from zymodemes that showed the biggest diversity were those from Z26, Z27, Z45 and Z74, all of them with 49 SNPs (Fig 6A). Based on this comparison, no correlation could be established between overall genetic divergence between the different isolates, evaluated from the previous phylogenetic analyses based on the genome sequences, and the variation observed for the genes encoding the selected enzymes used for MLEE.

**Table 1:** Metabolic enzymes whose genes were investigated for the SNP analysis from the ten *L*. *braziliensis* genomes sequenced here.

Initials	Enzymes	Gene ID
ICD	Isocitrate dehydrogenase	LbrM.33.2820
GPI	Glucose phosfate isomerase	LbrM.12.0490
ACON	Aconitate Hydratase	LbrM.18.0560
MDH	Malate dehydrogenase	LbrM.20.0030
G6PDH	Glucose -6-phosphate 1-dehydrogenase	LbrM.20.0160
PGM	Phospho glucomutase	LbrM.21.0700
ACP	Acide phosfatase	LbrM. 23.1290
FUM	Fumarase	LbrM.24.0320
ME	Malic enzyme	LbrM.24.0780
NH	Nucleoside Hydrolase	LbrM.29.2850
HK	Homoserine Kinase	LbrM.30.3070
MPI	Manose Phosphatase Isomerase	LbrM.32.1750
6PGDH	6-phosphogluconate dehydrogenase	LbrM.34.3250
NADP	Isocitrate dehydrogenase mitochondrial	LbrM.10.0310

We also evaluated the impact of the SNPs identified on individual enzymes and attempted to correlate it with previous variations in zymodeme profiles, as previously defined [29]. When the variations from the 10 isolates were summed up (Fig 6B), the Malic Enzyme (ME) was the one with the overall highest number of SNPs (70) and this variation is compatible with the zymodeme analysis showing also the most diverse profile for this enzyme and grouping both the Z72 and Z75 zymodemes. In contrast, the enzyme Acid Phosphatase (ACP) did not show any variation for the samples studied, while in the zymodeme data this enzyme was associated with two distinct profiles. More relevant was the identification of a single SNP for the enzyme Glucose-6-Phosphate Dehydrogenase (G6PDH), common to all of the isolates sequenced here (Fig 6B), while five different profiles were reported for this enzyme in the zymodeme analysis, with this profile indeed defining the *L*. *shawi* Z26 zymodeme. Also the enzyme Aconitate Hydratase (ACON) was found in our analysis to be the one with the highest number of SNPs (9) for the Z72 and Z75 isolates, with tree to seven SNPs also seen for the other isolates, but no variation was seen in the zymodeme profile for this enzyme for any of the previously evaluated isolates. The conclusion from these results then is that phylogenetic inferences derived from the MLEE analysis are very limited at least when comparing closely related isolates or species. The genetic data does not necessarily reflect the zymodeme profiles and other factors, perhaps post-translational modifications differentially targeting one or more of the enzymes, might be interfering with their electrophoresis migration and altering these profiles.

### Ploidy number definition

**Fig 7:**
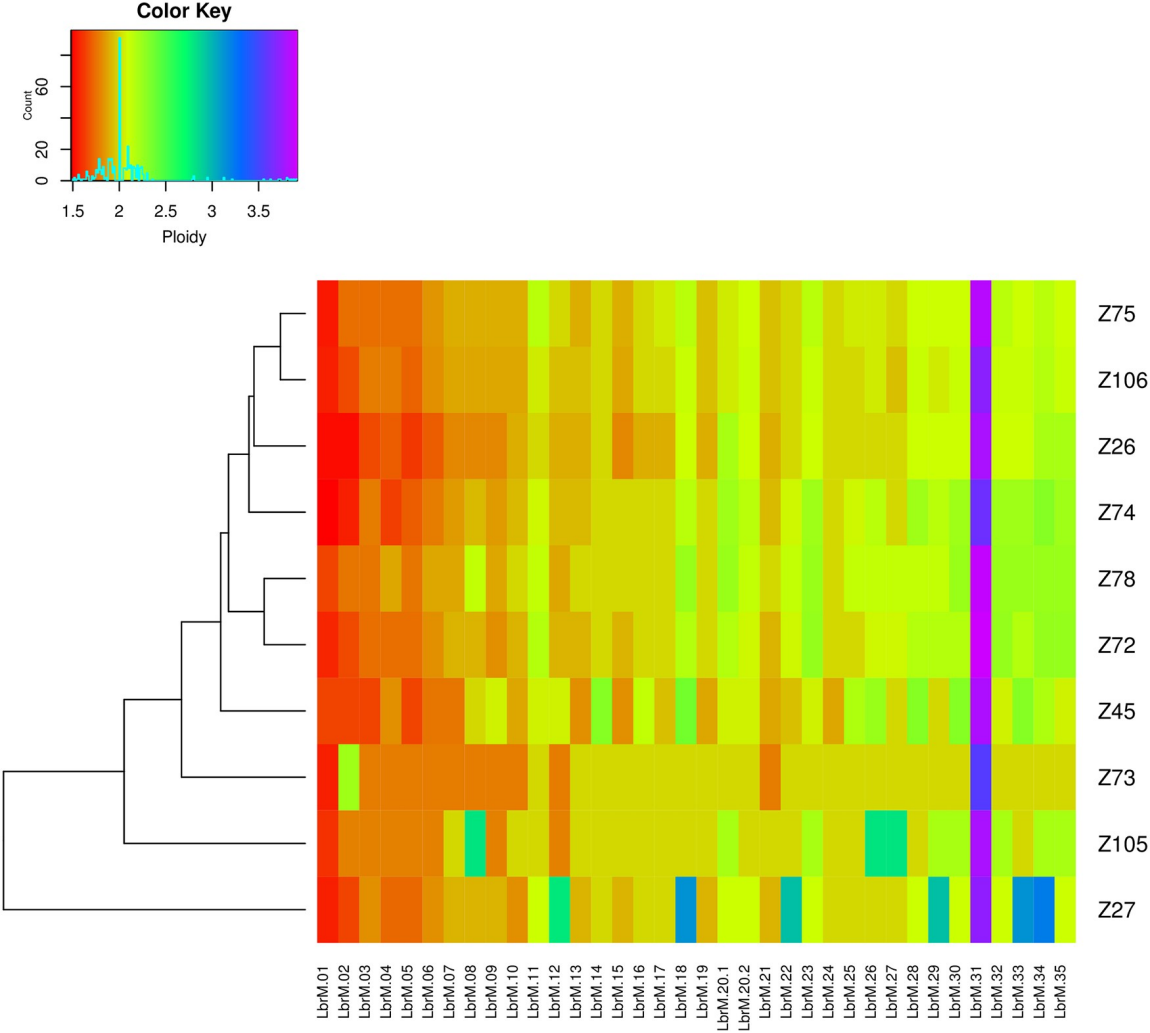
Chromosome copy number evaluation. Ploidy in natural population of *L*. *braziliensis*. The heatmap shows the copy-number status for all chromosomes from the ten genomes studied here. Based on the histogram present in the color key (top left), it is possible to notice that the majority of chromosomes are predicted to have two copies, with an average ploidy of 2. See Material and methods for ploidy estimation.

It has been suggested that variations in the number of gene copies in *Leishmania* may affect gene expression, which may also contribute to the adaptation and tropism of this parasite [55, 56]. It is likely that gene dosage may play an important role in the regulation of expression, given the apparent lack of other mechanisms of transcriptional regulation in trypanosomatids, and this may be associated with changes in chromosomal copy numbers. In the present study, we used the sequenced reads to estimate chromosome copy number for all sequenced isolates and investigated possible aneuploidy events. For nearly all isolates sequenced here, a diploid number for the majority of the chromosomes was predicted, with the consistent exception of chromosome 31 (Fig 7). The single exception among the isolates was the one from zymodeme Z27 which presented six different chromosomes (12, 18, 22, 29, 33 and 34) with likely extra chromosomal copies. Chromosome 31 was represented by more than three copies in all ten isolates; consistent with this chromosome being supernumerary in all *Leishmania* species sequenced so far [10]. Overall our results are consistent with a limited occurrence of aneuploidy in the isolates investigated, and presumably in native *L*. *braziliensis* from the corresponding areas, with this being mainly restricted to chromosome 31.

### Heterozygosity estimation

**Fig 8:**
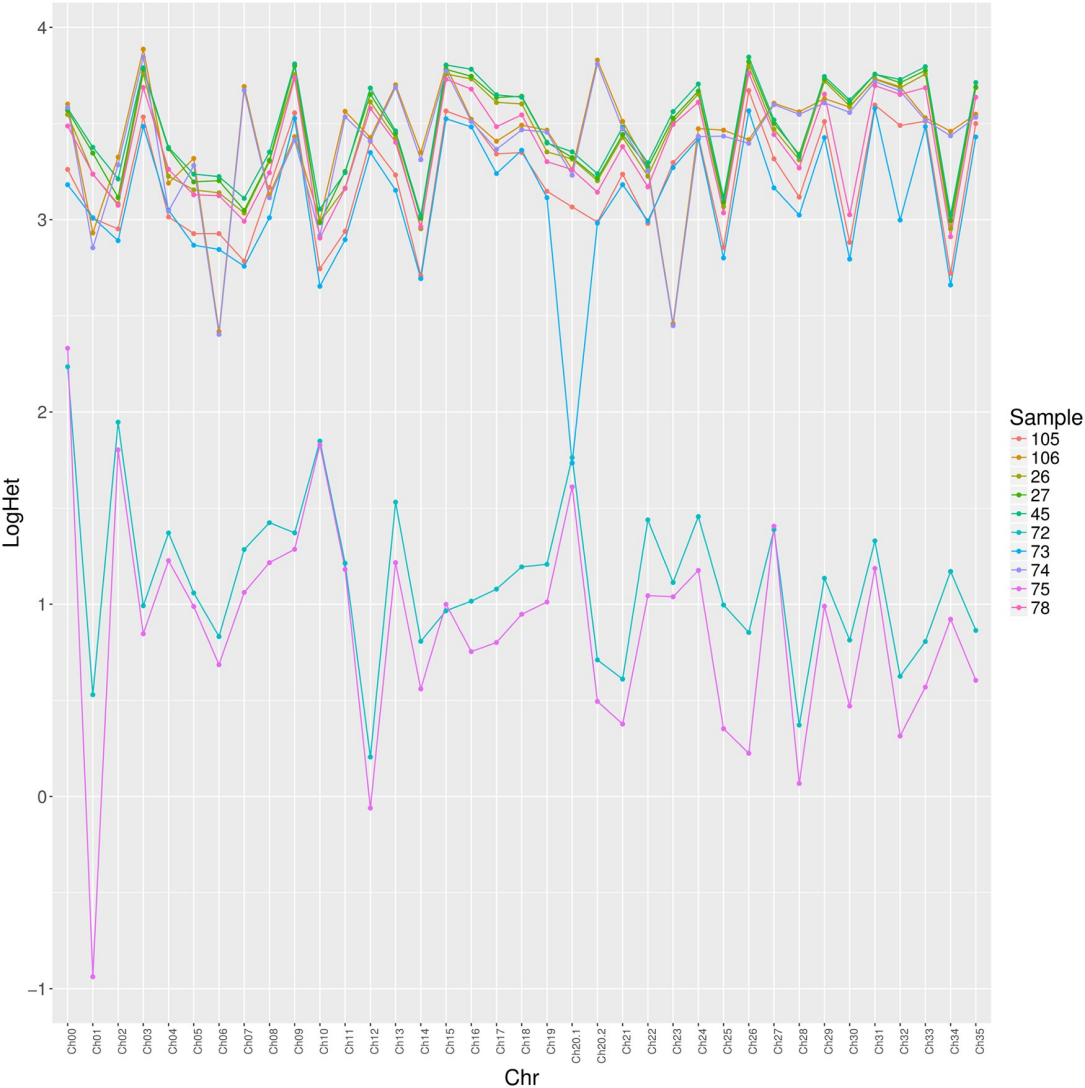
Heterozygosity plot for each chromosome of the ten *L*. *braziliensis* genomes investigated in this study.

Previous estimates of *L*. *braziliensis* heterozygosity have led to contrasting results, with a first report indicating low levels of heterozygosity in selected populations through the study of polymorphic microsatellite loci [57], followed by a whole genome analysis finding much greater heterozygosity in *L*. *braziliensis* when compared to *L*. *major* or *L*. *infantum* [10]. One possibility for the low heterozygosity might be extensive inbreeding through sexual recombination and preferential mating between closely related isolates. In a larger time scale this could lead to genetic diversification, as seen here between the different *L*. *braziliensis* groups. So, in order to evaluate if changes in heterozygosity could be identified between the various isolates investigated in this study, the SNP data derived from the sequencing effort was used to calculate the percentage of heterozygosity for the identified SNPs within each of the ten genomes described here. A comparison of the heterozygosity values derived from each chromosome for all ten genomes is shown in Fig 8. Two contrasting patterns are observed, with the two isolates from zymodemes Z72 and Z75 having very low heterozygosity in all chromosomes while all other isolates, including the pair from zymodemes Z74 and Z106, having much higher levels of heterozygosity, again in all chromosomes. These results are once more in agreement with the clustering of the Z72 and Z75 isolates as a genetically distinct *L*. *braziliensis* group. It also reinforces the lack of genetic exchange between these isolates and more genetically diverse *L*. *braziliensis*. In contrast, the Z74 and Z106 isolates, despite also clustering as a separate group, do not to seem to be as restricted. These results again reinforce the so far unnoticed complexity in the genetic structure of the *L*. *braziliensis* isolates investigated here.

## Discussion

This study highlights the largely diverse nature of *L*. *braziliensis*, a diversity seen even within restricted or adjacent areas and that may reflect the complexity in vertebrate hosts and vectors responsible for the maintenance and transmission of CL. In Pernambuco, where *L*. *braziliensis* is the predominant etiological agent of this disease, various phlebotomine sandfly species are widely distributed over the state’s territory and several small rodents have been incriminated as major reservoirs hosts, with *Nectomys squamipes*, *Necromys lasiurus* and *Rattus rattus* proposed as hosts for the peridomestic zoonotic cycle of CL [31, 33, 58, 59]. In the municipality of Paudalho, from where the Z72 and Z75 isolates were retrieved, the most abundant vectors are *Lutzomyia complexa* and *Lutzomyia choti*, predominantly involved in the parasite life cycle in forest remnants. For the other two municipalities investigated here, Amaraji and Moreno, the domestic and peri-domestic cycles of CL are well established and *Lutzomyia whitmani* is incriminated as a vector, being well adapted to the urbanized environment [30–33]. Our data is consistent with the different hosts/vectors being linked with the distinct grouping of the Z72 and Z75 isolates and it may also explain the lack of heterozygosity associated with these parasites, perhaps due to the isolation within the restricted forest environment.

In a pioneering way, the sequencing, mapping and variant annotation of the ten genomes described here have clarified the genetic relatedness of divergent isolates from neighboring areas in Brazil, raising major clues regarding the ecology and transmission patterns of *L*. *braziliensis*, and ruling out aneuploidy as a major source of genetic variation in these isolates. More recently, studies based on different genomes from *Leishmania* and related organisms have added important information regarding resistance to treatment, mechanisms of adaptation and targets for new drugs, emphasizing the advantages of complete genomic analysis to clarify vital processes of these parasites [50, 60–62]. The genome sequencing, using high-throughput sequencers, generates large amounts of data in single runs, enabling a broader analysis of the targeted isolates. Nevertheless, our results show that the markers used in the phylogenetic analysis, the four metabolic enzymes and Hsp70, generated similar results when compared to the whole genome analysis, again showing the strength of these markers in the study of *Leishmania* variability. Indeed, the three evolutionary groups of *L*. *braziliensis* identified here were confirmed by phylogenetic trees based on these markers, as well as on the whole genomes, and these are in agreement with studies already published, which highlight the importance of these markers, both for characterization, as well as in phylogenetic studies [42, 43, 63]. In contrast, the disagreement between the genetic variability and the phenotypic classification of these isolates made by the MLEE highlights the need to replace the enzyme analysis by a Multilocus Sequence Analysis (MLSA) for the identification of strains and isolates, as has been previously proposed Boité et al. [42], or even whole genome sequencing, when dealing with more closely related ones. Indeed, the markers ICD, MPI, 6PGDH and G6PDH are powerful enough to discriminate between different species of the subgenus *Viannia* including *L*. (*V*.) *lindenbergi*, *L*. (*V*.) *lainsoni*, *L*. (*V*.) *naiffi* and *L*. (*V*.) *guyanensis* [42,64]. In addition, they also have intra-specific discriminatory power when comparing more distantly related isolates/strain, as demonstrated here and in the study by Marlow et al., 2014 [65], which used the 6PGD, MPI and Hsp70 genes to answer epidemiological questions involving genetic groups of *L*. *braziliensis*. It is evident that MLEE may contribute to the characterization of *Leishmania* sp., but it does not provide relevant information regarding intra-specific variations.

Aneuploidy has been considered one of the most important processes for the adaptation of *Leishmania* and is also related to drug resistance since it is an alternative for the recombination of genotypes [50, 52, 56, 62, 66]. Several studies have revealed the variations in the number of chromosome copies in this trypanosomatid, indicating that aneuploidy is a constitutive characteristic that seems beneficial in a unicellular organism that is primarily asexual. Indeed aneuploidy has been confirmed in several species of *Leishmania* (*L*. *braziliensis*, *L*. *donovani*, *L*. *infantum*, *L*. *major* and *L*. *mexicana*), but the number of copies varied considerably. The extra number of copies for chromosome 31 in different *Leishmania* species is more consistent, but it is not clear why this chromosome is specifically targeted by the greatest numerical changes [10, 67]. Our findings corroborate the extra number of copies for chromosome 31, however it does not support extensive aneuploidy as source of variation between different isolates, at least as observed for *L*. *braziliensis*.

Another important aspect that must be taken into account deals with the reproduction strategies in *Leishmania* that lead to evolutionary processes, generating discussions since the 1990s on the asexual versus sexual reproduction of these parasites [24, 57,68, 69]. Intra-specific sexual recombination has been subsequently confirmed in *Leishmania*, with the meiotic cell cycle linked with the invertebrate host [70]. It is possible then that the differences in heterozygosity observed here may reflect differences in the extent of sexual recombination occurring within the different groups identified. These differences need to be more properly investigated in the future and their causes defined.

Overall we observed the possibility of three distinct phylogenetic groups of *L*. *braziliensis* in the state of Pernambuco, which proves that the transmission profile, involving several vectors, directly interferes with the genetic pattern of the circulating lineages. Our results specifically highlight the differences between isolates originating from forested environments and isolates recovered from patients who became infected in urbanized environments. The Atlantic Forest region represents more than 60% of all CL cases reported in Pernambuco, which emphasizes the need to establish preventive measures to the disease. The finding of heterogeneity of *L*. *braziliensis* in this area strongly reinforces previous evidences for the complexity of its transmission cycle.

## Supporting information

S1 Table Summary of the alignment metrics for the 10 genomes of *L*. *braziliensis* described here. Generated by the Picard tool (V. 1.117). Pf- Pass Filter, HQ- High quality.
(DOCX)

S1 Fig Assessment of the quality generated by the sequenced reads. **A**. Mean quality score. **B**. Per sequence quality score.
(TIF)

S2 Fig Assessment of the sequenced depth for all chromosomes from the sequenced samples. Mean depths for the readings from the whole genome of the 10 sequenced *L*. *braziliensis* isolates. Ten kilobase windows were used, with the y-axis plotted logarithmically.
(TIF)
