# Supplementary material for: Identification of divergent Leishmania (Viannia) braziliensis ecotypes derived from a geographically restricted area through whole genome analysis
Source: PLoS Negl Trop Dis. 2019 Jun 6;13(6):e0007382. doi: 10.1371/journal.pntd.0007382 (PMC6581274; doi:10.1371/journal.pntd.0007382)
Supplement: S1 Table — Generated by the Picard tool (V. 1.117). Pf- Pass Filter, HQ- High quality. (DOCX) [file pntd.0007382.s001.docx]

**Supporting information**

**Table S1. Summary of the alignment metrics for the 10 genomes of *L. braziliensis* described here.**

Generated by the Picard tool (V. 1.117).

|  | Reads | | | | | Bases | |
| --- | --- | --- | --- | --- | --- | --- | --- |
| Strain | Total | Pf_Aligned | %_Aligned | Pf_HQ_Aligned ( ≥ Q20) | Mean_Length | Pf_Aligned | Pf_HQ_Aligned |
| Z26 | 2494132 | 2271177 | 0.910608 | 2175338 | 210.085979 | 445346789 | 428647651 |
| Z27 | 3026084 | 2807932 | 0.927909 | 2691354 | 211.477861 | 563544139 | 542651753 |
| Z45 | 4047320 | 3824431 | 0.944929 | 3658457 | 205.412069 | 751484264 | 722064457 |
| Z72 | 2759292 | 2673651 | 0.968963 | 2559452 | 203.356989 | 529229436 | 508572177 |
| Z73 | 1028976 | 1000624 | 0.972446 | 956219 | 188.32297 | 184426184 | 176945650 |
| Z74 | 3232156 | 3084309 | 0.954257 | 2961213 | 208.875645 | 617569129 | 595187713 |
| Z75 | 4434870 | 4295639 | 0.968605 | 4103939 | 207.919081 | 869026099 | 833632947 |
| Z78 | 2013112 | 1981848 | 0.984470 | 1899433 | 206.772345 | 404370949 | 388835479 |
| Z105 | 1166600 | 1146860 | 0.983079 | 1098604 | 207.58792 | 234535353 | 225502082 |
| Z106 | 5116622 | 4554467 | 0.890132 | 4351343 | 211.022068 | 860770673 | 827062517 |

Pf- Pass Filter, HQ- High quality
