# Supplementary figures and images for: Identification of divergent Leishmania (Viannia) braziliensis ecotypes derived from a geographically restricted area through whole genome analysis
Source: PLoS Negl Trop Dis. 2019 Jun 6;13(6):e0007382. doi: 10.1371/journal.pntd.0007382 (PMC6581274; doi:10.1371/journal.pntd.0007382)

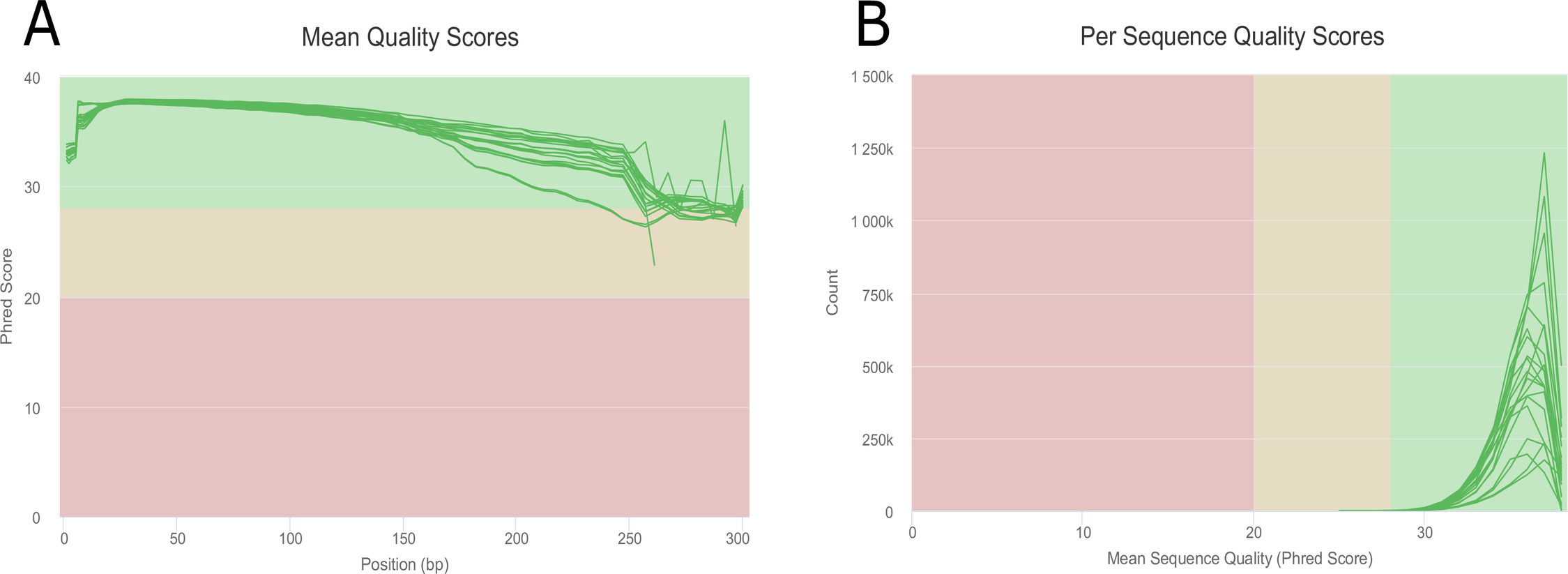

Supplement: S1 Fig — A. Mean quality score. B. Per sequence quality score. (TIF) [file pntd.0007382.s002.tif]

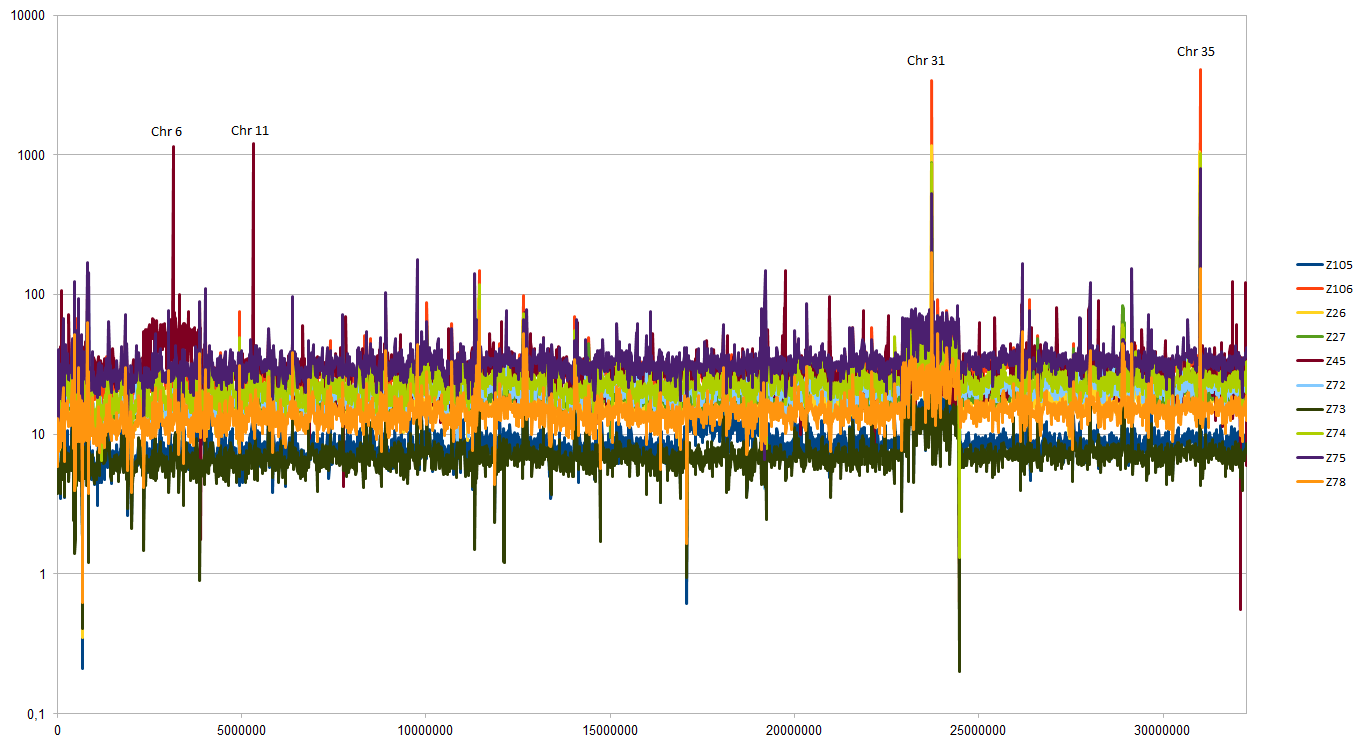

Supplement: S2 Fig — Mean depths for the readings from the whole genome of the 10 sequenced L. braziliensis isolates. Ten kilobase windows were used, with the y-axis plotted logarithmically. (TIF) [file pntd.0007382.s003.tif]
